# Factors associated with the presentation of erosive esophagitis symptoms in health checkup subjects: A prospective, multicenter cohort study

**DOI:** 10.1371/journal.pone.0196848

**Published:** 2018-05-03

**Authors:** Naomi Mochizuki, Tsuyoshi Fujita, Masao Kobayashi, Yukinao Yamazaki, Shuichi Terao, Tsuyoshi Sanuki, Akihiko Okada, Masayasu Adachi, Manabu Murakami, Yoshifumi Arisaka, Koji Uno, Atsuhiro Masuda, Masaru Yoshida, Eiji Umegaki, Hiromu Kutsumi, Takeshi Azuma

**Affiliations:** 1 Department of Health Care, Kyoto Second Red Cross Hospital, Kyoto, Kyoto, Japan; 2 Department of Health Care, Yodogawa Christian Hospital, Osaka, Osaka, Japan; 3 Department of Gastroenterology, Fukui Red Cross Hospital, Fukui, Fukui, Japan; 4 Department of Gastroenterology, Kakogawa Central City Hospital, Kakogawa, Hyogo, Japan; 5 Department of Gastroenterology, Kita-harima Medical Center, Ono, Hyogo, Japan; 6 Department of Gastroenterology, Saiseikai Nakatsu Hospital, Osaka, Osaka, Japan; 7 Hotel Okura Kobe Clinic, Kobe, Hyogo, Japan; 8 Division of Gastroenterology, Department of Internal Medicine, Kobe University Graduate School of Medicine, Kobe, Hyogo, Japan; 9 Department of Gastroenterology, Nissay Hospital, Osaka, Osaka, Japan; 10 Department of Gastroenterology, Kyoto Second Red Cross Hospital, Kyoto, Kyoto, Japan; 11 Center for Clinical Research and Advanced Medicine, Shiga University Medical Science, Otsu, Shiga, Japan; University Hospital Llandough, UNITED KINGDOM

## Abstract

**Background:**

We aimed to clarify the factors associated with the presentation of erosive esophagitis (EE) symptoms in subjects undergoing health checkups.

**Methods:**

We utilized baseline data from 7,552 subjects who underwent upper endoscopy for health screening in a prospective, multicenter cohort study. The subjects were asked to complete a questionnaire detailing their upper abdominal symptoms and lifestyle. Based on the heartburn and/or acid regurgitation frequency, the EE subjects were stratified into the following three groups: (1) at least one day a week (symptomatic EE [sEE]), (2) less than one day a week (mild symptomatic EE [msEE]), and (3) never (asymptomatic EE [aEE]). Postprandial distress syndrome (PDS) and epigastric pain syndrome (EPS) were defined according to the Rome III criteria.

**Results:**

Of the 1,262 (16.7%) subjects (male 83.8%, mean age 52.6 years) with EE, the proportions of sEE, msEE and aEE were 15.0%, 37.2% and 47.9%, respectively. The sEE group showed significant associations with overlapping EPS (OR: 58.4, 95% CI: 25.2–160.0), overlapping PDS (OR: 9.96, 95% CI: 3.91–26.8), severe hiatal hernia (OR: 2.43, 95% CI: 1.43–4.05), experiencing high levels of stress (OR: 2.20, 95% CI: 1.43–3.40), atrophic gastritis (OR: 1.57, 95% CI: 1.03–2.36) and Los Angeles (LA) grade B or worse (OR: 1.72, 95% CI: 1.12–2.60) in the multivariate analysis.

**Conclusions:**

Approximately one-sixth of EE subjects were symptomatic. A multifactorial etiology, including factors unrelated to gastric acid secretion, was associated with the symptom presentation of EE subjects.

## Introduction

Gastroesophageal reflux disease (GERD) is a condition that develops when the reflux of stomach contents causes troublesome symptoms and/or complications [1]. GERD is prevalent worldwide [2], and the prevalence of GERD in Japan has been increasing since the end of the 1990s [3,4].

GERD is divided into two groups according to endoscopic findings: (1) erosive esophagitis (EE) and (2) nonerosive gastroesophageal reflux disease (NERD). The EE prevalence is 15.5% in the Swedish adult population [5] and 12.4% in Spanish patients who have undergone upper gastrointestinal endoscopy [6]. In Asia, including Japan, the prevalence of EE ranges from 4.5% to 15.7% [3, 7]. GERD symptoms are not always associated with the presence or absence of esophagitis, and asymptomatic EE is common. In a study of the adult Swedish population, 37% of EE cases were asymptomatic [5]. The nationwide Japanese GERD survey reported that 210 of 600 (35%) patients with EE were asymptomatic [8]. Similarly, recent studies from other Asian countries conducted in subjects undergoing health checkups have reported that approximately half or more of EE patients are asymptomatic [9–11]. The symptom presentation of EE patients is clinically important because GERD symptoms reduce patient quality of life (QOL) [12,13]. However, the factors associated with the presentation of EE symptoms have not been fully elucidated.

The present study aimed to prospectively investigate the prevalence of EE in a large population undergoing health checkups at 7 facilities and to determine the factors associated with the symptom manifestation of EE, which lowers patient QOL.

## Materials and methods

### Subjects

This study was based on baseline data (n = 8,889) from the Upper Gastro Intestinal Disease (UGID) study currently being conducted in Japan. The UGID study is a prospective, multicenter cohort study that aims to investigate the prevalence and natural history of EE, NERD and functional dyspepsia (FD). In the UGID study, subjects 18 years of age or older who underwent upper endoscopy for health screenings at 7 facilities will be followed for up to 5 years. This study population includes 7,552 subjects who were enrolled between April 2013 and March 2015. The following exclusion criteria were applied: treatment with a proton pump inhibitor (PPI) or histamine type 2-receptor antagonist (H2RA) (n = 597), incomplete data for endoscopic findings (n = 59), reflux symptoms or the dyspeptic symptom questionnaire (n = 79), smoking (n = 25), consumption of alcohol (n = 24), kyphosis (n = 112), and State-Trait Anxiety Inventory (STAI) traits (n = 441). This study was conducted in accordance with the Declaration of Helsinki and its amendments (UMIN-CTR ID: 000022504). The study protocol was approved by the ethics committee at each institution (the ethics committees of Kyoto Second Red Cross Hospital, Yodogawa Christian Hospital, Fukui Red Cross Hospital, Kakogawa Central City Hospital, Kita-harima Medical Center, Saiseikai Nakatsu Hospital and Hotel Okura Kobe Clinic). Written informed consent was obtained from all study subjects. All authors had access to the study data and reviewed and approved the final manuscript.

### Questionnaire

The subjects were asked to complete the questionnaire about their upper abdominal symptoms (reflux symptoms, dyspeptic symptoms), height, body weight, smoking (never, ex-smoker, current smoker), alcohol consumption (none, ≤20 g/day, 20–60 g/day or >60 g/day), sleep shortage (yes, no), exercise shortage (yes, no), irregular meal times (yes, no), experiencing high levels of stress (yes, no), feeling depressed (yes, no), and kyphosis. For reflux symptoms, the frequency of heartburn and/or acid regurgitation in past 3 months was investigated (i.e., never, less than one day a month, one day a month, two to three days a month, one day a week, more than one day a week, or every day). For dyspeptic symptoms of postprandial distress syndrome (PDS), the frequency of bothersome postprandial fullness and/or early satiation in the past 3 months was investigated (i.e., never, less than one day a month, one day a month, two to three days a month, one day a week, more than one day a week, or every day). For dyspeptic symptoms of epigastric pain syndrome (EPS), the frequency of epigastric pain and/or epigastric burning experienced in the past 3 months was investigated (i.e., never, less than one day a month, one day a month, two to three days a month, one day a week, more than one day a week, or every day). In addition, the subjects were asked whether the onset of dyspeptic symptoms occurred more than 6 months ago. The presence or absence of "sleep shortage", "exercise shortage", "irregular meal times", "experience of high levels of stress" and "feeling depressed" was investigated without the inclusion of objective definitions in this self-reported questionnaire survey. Kyphosis was assessed with the following question. “Does the back of your head touch the wall when you stand with your heels touching the wall?” The possible answers were “Yes, easily”, “Yes but not easily”, or “No”. The subjects who answered “No” were defined as positive for kyphosis, as diagnosed through the questionnaire.

Anxiety was assessed based on the STAI score [14]. The STAI (trait) inventory consists of twenty questions, and higher scores indicate stronger anxiety. A high STAI score was defined as ≥44 in male subjects and ≥45 in female subjects.

The medications that the subjects were prescribed were investigated by the health screening questionnaire used at each institution. In this study, the use of PPI (presence, absence), H2RA (presence, absence), other gastromucoprotective agents (presence, absence), non-steroidal anti-inflammatory drugs (NSAIDs) (presence, absence), low-dose aspirin (presence, absence), calcium (Ca) antagonists (presence, absence), angiotensin II receptor blockers (ARBs) (presence, absence), statins (presence, absence), oral antidiabetic agents (presence, absence), and bisphosphonate (presence, absence) was investigated.

### Endoscopic findings

EE was diagnosed based on the presence of endoscopically detectable mucosal breaks and was graded according to the Los Angeles (LA) classification. The presence of endoscopic Barrett’s mucosa was defined by a greater than 10-mm length of the endoscopic columnar-lined epithelium, which was diagnosed using the palisade vessels as a landmark for the esophagogastric junction [15]. Hiatal hernia was diagnosed based on proximal dislocation of the esophagogastric junction of more than 2 cm above the diaphragmatic hiatus. Hiatal hernia severity was classified by the length of dislocation of the esophagogastric junction; 2–4 cm was considered mild, while >4 cm was considered severe. Atrophic gastritis was endoscopically diagnosed, and the endoscopic extent of atrophic mucosa was graded according to the Kimura–Takemoto classification from C-1 to O-3 [16]. Subjects with atrophic mucosa (graded as C-2, C-3, O-1, O-2, and O-3) were defined as positive for atrophic gastritis.

### Definitions of symptomatic EE, mild symptomatic EE, asymptomatic EE, NERD, and the control group

The EE subjects who had neither peptic ulcers nor any history of upper gastrointestinal tract surgery were classified into one of three groups according to the frequency of reflux symptoms: the symptomatic EE (sEE) group (subjects with heartburn and/or acid regurgitation occurring at least one day a week), mild symptomatic EE (msEE) group (subjects with heartburn and/or acid regurgitation occurring less than one day a week), or asymptomatic EE (aEE) group (subjects with neither heartburn nor acid regurgitation). Subjects with heartburn and/or acid regurgitation occurring at least one day a week, without EE, peptic ulcers or any history of upper gastrointestinal tract surgery, were defined as having NERD. Subjects with no heartburn, acid regurgitation, bothersome postprandial fullness, early satiation, epigastric pain or epigastric burning, and without EE, peptic ulcer, upper gastrointestinal tract malignancy, or a history of upper gastrointestinal tract surgery were defined as the control group.

### Definitions of FD, PDS and EPS

The FD group was defined as those subjects who experienced PDS and/or EPS, according to the Rome III criteria [17]. PDS was defined as "bothersome postprandial fullness" and/or "early satiation" occurring at least two days a week in the past 3 months, with symptom onset occurring 6 months ago, and EPS was defined as "epigastric pain" or "epigastric burning " occurring at least one day a week in the past 3 months, with an onset of symptoms occurring 6 months ago.

In this study, the GERD subjects were not excluded from the FD group.

### Statistical analyses

All statistical analyses were conducted using JMP version 10 (SAS Institute, Cary, NC, USA). Comparisons between the subjects with EE and the control group as well as between the subjects with sEE and the subjects with msEE plus aEE were performed with the χ^2^ test (or the Fisher exact test, if appropriate) for categorical variables and Student’s *t*-test for continuous variables. Categorical variables, including age, gender, current smoking status, alcohol consumption ≥20 g/day, and variables with *P* values less than 0.2 (according to the χ^2^ test) were used for the multiple logistic regression analyses, and *P*<0.05 was considered statistically significant.

## Results

### Prevalence and factors associated with EE

Of the 7,552 study subjects, 1,262 subjects (16.7%) (1,058 males and 204 females, mean age±SD: 52.6±9.4 years) were found to have EE. Most cases of EE were of mild severity (LA grade A, 79.7%, and LA grade B, 18.0%) (Table 1).

**Table 1:** Characteristics of the study participants.

	Total (n = 7,552)
Age, mean±SD (years)	52.4±10.0
Age group	
≤39 years	715 (9.5%)
40–59 years	4,997 (66.2%)
≥60 years	1,840 (24.4%)
Gender	
Men	4,766 (63.1%)
Women	2,786 (36.9%)
Body mass index, mean±SD (kg/m^2^)	22.9±3.3
Body mass index ≥25 kg/m^2^	1,747 (23.1%)
Current smoking	1,247 (16.5%)
Alcohol consumption ≥20 g/day	2,027 (26.8%)
Self-assessment on the daily life questionnaire	
Sleep shortage	2,480 (32.8%)
Exercise shortage	4,814 (63.7%)
Irregular meal time	1,597 (21.1%)
Experiencing high levels of stress	2,099 (27.8%)
Feeling depressed	671 (8.9%)
Kyphosis diagnosed by questionnaire	39 (0.52%)
STAI score, mean±SD	41.6±9.9
High STAI score	2,867 (38.0%)
Endoscopic findings	
Atrophic gastritis	2,949 (39.0%)
Hiatal hernia	2,212 (29.3%)
Mild	1,934/2,212 (87.4%)
Severe	278/2,212 (12.6%)
Endoscopic Barret’s mucosa ≥10 mm	160 (2.1%)
Erosive esophagitis (EE)	1,262 (16.7%)
LA grade A	1,006/1,262 (79.7%)
LA grade B	227/1,262 (18.0%)
LA grade C	27/1,262 (2.1%)
LA grade D	2/1,262 (0.16%)
Symptomatic EE	189/1,262 (15.0%)
Mild symptomatic EE	469/1,262 (37.2%)
Asymptomatic EE	604/1,262 (47.9%)
NERD	363 (4.8%)
FD	299 (4.0%)
PDS	170 (2.3%)
EPS	183 (2.4%)
Control group	3,254 (43.1%)
Current medication	
NSAIDs	122 (1.6%)
Low-dose aspirin	89 (1.2%)
Ca antagonists	702 (9.3%)
ARB	602 (8.0%)
Statins	717 (9.5%)
Oral hypoglycemic agents	251 (3.3%)
Bisphosphonate	34 (0.45%)
Gastromucoprotective agents	176 (2.3%)

*SD*, standard deviation; *STAI*, State-Trait Anxiety Inventory; *NERD*, nonerosive reflux disease; *FD*, functional dyspepsia; *PDS*, postprandial distress syndrome; *EPS*, epigastric pain syndrome; *NSAIDs*, non-steroidal anti-inflammatory drugs; *Ca*, calcium; *ARB*, angiotensin II receptor blocker.

**Fig 1:**
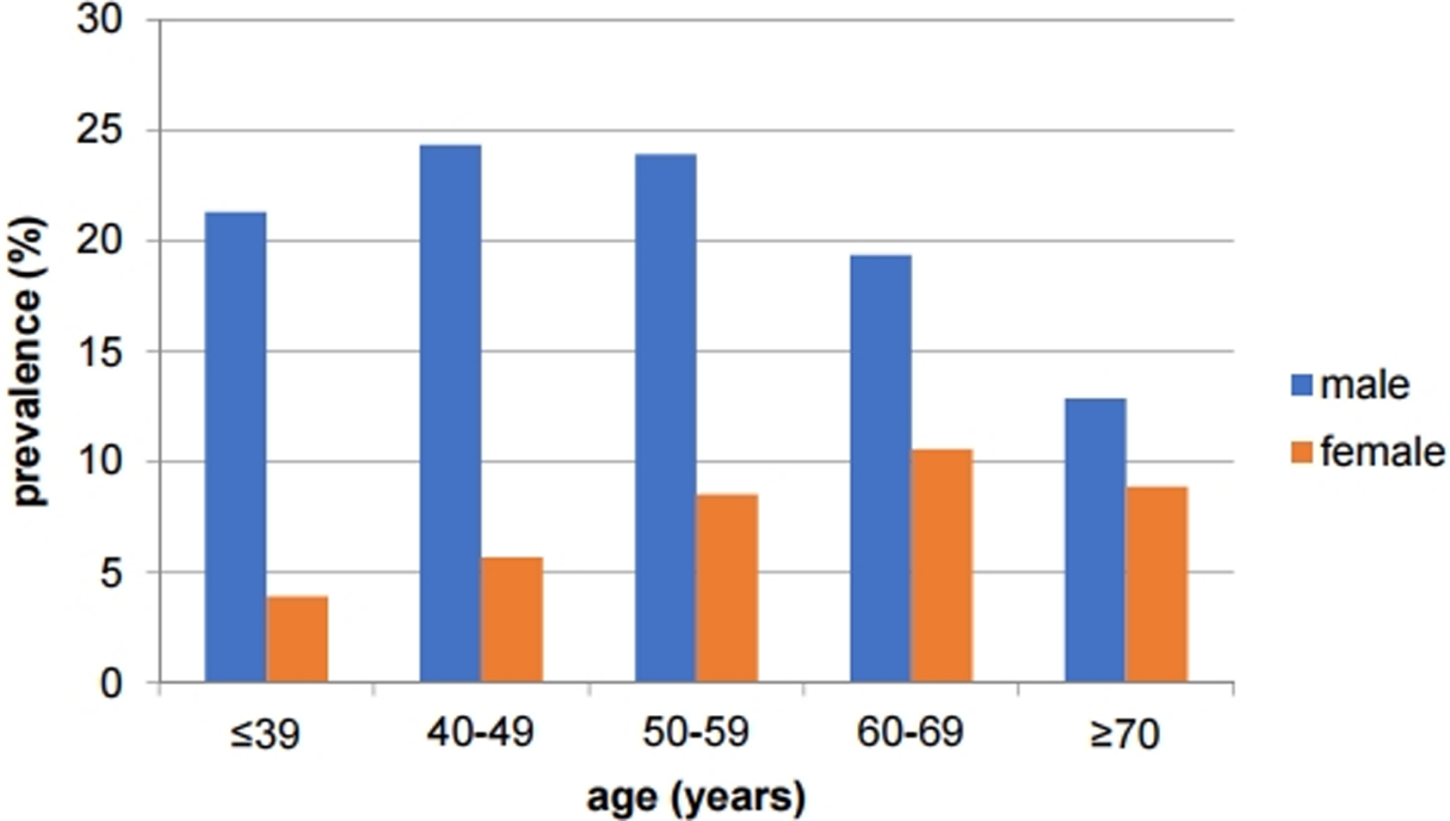
Age and gender distributions for the prevalence of erosive esophagitis. At all ages, male subjects were diagnosed with erosive esophagitis more frequently than female subjects.

Fig 1 shows the age and gender distribution for the prevalence of EE. At all ages, male subjects were diagnosed with EE more frequently than female subjects (22.2% in males vs. 7.3% in females, *P*<0.0001), although the difference was less pronounced in the elderly (12.9% in males over 70 years old vs. 8.9% in females over 70 years old, *P* = 0.2497).

Comparisons of the clinical characteristics of the subjects with EE and the control group are shown in Table 2. Gender, body mass index (BMI), current smoking, alcohol consumption ≥20 g/day, exercise shortage, irregular meal time, experiencing high levels of stress, feeling depressed, STAI score, hiatal hernia, endoscopic Barrett’s mucosa ≥10 mm, atrophic gastritis, and the use of Ca antagonists, ARB, and oral hypoglycemic agents differed significantly between the EE group and the control group.

**Table 2:** Comparison of clinical characteristics between the subjects with erosive esophagitis and the control group.

	EE (n = 1,262)	Control (n = 3,254)	*P* value
Age, mean±SD (years)	52.6±9.4	52.7±10.3	0.5524
Age group			0.0037
≤39 years (%)	7.8	9.5	
40–59 years (%)	69.3	64.0	
≥60 years (%)	22.9	26.5	
Male (%)	83.8	61.7	<0.0001
BMI, mean±SD (kg/m^2^)	24.3±3.6	22.7±3.1	<0.0001
BMI ≥25 kg/m^2^ (%)	37.0	20.3	<0.0001
Current smoking (%)	24.9	14.8	<0.0001
Alcohol consumption ≥20 g /day (%)	38.9	23.7	<0.0001
Sleep shortage (%)	30.0	28.4	0.3001
Exercise shortage (%)	65.1	60.8	0.0076
Irregular meal time (%)	22.7	19.2	0.0074
Experiencing high levels of stress (%)	28.0	20.6	<0.0001
Feeling depressed (%)	7.1	5.5	0.0419
Kyphosis diagnosed by questionnaire (%)	0.71	0.34	0.0885
STAI score, mean±SD	41.1±9.9	40.0±9.5	0.0004
High STAI score (%)	37.4	31.3	<0.0001
Hiatal hernia (%)	48.3	24.4	<0.0001
Endoscopic Barret’s mucosa ≥10 mm (%)	4.5	1.4	<0.0001
Atrophic gastritis (%)	24.5	40.8	<0.0001
Use of NSAIDs (%)	1.7	1.2	0.2561
Use of low-dose aspirin (%)	0.87	1.4	0.1636
Use of Ca antagonists (%)	12.5	9.0	0.0005
Use of ARB (%)	10.1	7.3	0.0018
Use of statins (%)	10.7	9.3	0.1678
Use of oral hypoglycemic agents (%)	5.1	3.0	0.0010
Use of bisphosphonate (%)	0.16	0.40	0.2606
Use of gastromucoprotective agents (%)	2.6	2.0	0.2270

*EE*, erosive esophagitis; *SD*, standard deviation; *BMI*, body mass index; *STAI*, State-Trait Anxiety Inventory; *NSAIDs*, non-steroidal anti-inflammatory drugs; *Ca*, calcium; *ARB*, angiotensin II receptor blocker.

The results of the multivariate analysis of factors associated with EE indicated that an age of 40–59 years (reference: ≤39 years) (OR 1.44, 95% CI 1.12–1.88), age of ≥60 years (reference: ≤39 years) (OR 1.49, 95% CI 1.10–2.03), male gender (OR 2.32, 95% CI 1.93–2.80), BMI ≥25 kg/m^2^ (OR 1.87, 95% CI 1.60–2.19), current smoking status (OR 1.34, 95% CI 1.12–1.61), alcohol consumption ≥20 g/day (OR 1.57, 95% CI 1.34–1.84), experiencing high levels of stress (OR 1.40, 95% CI 1.17–1.68), hiatal hernia (OR 2.41, 95% CI 2.08–2.79), endoscopic Barret’s mucosa ≥10 mm (OR 2.62, 95% CI 1.71–4.04), atrophic gastritis (OR 0.40, 95% CI 0.34–0.47), and use of low-dose aspirin (OR 0.38, 95% CI 0.17–0.76) were significantly different between the EE group and the control group (Table 3).

**Table 3:** Multivariate analysis of the factors associated with erosive esophagitis compared to the control group.

	OR	95% CI	*P* value
Age			
40–59 years (reference: ≤39 years)	1.44	1.12–1.88	0.0050
≥60 years (reference: ≤39 years)	1.49	1.10–2.03	0.0096
Gender (male/female)	2.32	1.93–2.80	<0.0001
BMI ≥25 kg/m^2^ (yes/no)	1.87	1.60–2.19	<0.0001
Current smoking (yes/no)	1.34	1.12–1.61	0.0012
Alcohol consumption ≥20 g /day (yes/no)	1.57	1.34–1.84	<0.0001
Experiencing high levels of stress (yes/no)	1.40	1.17–1.68	0.0003
Hiatal hernia (yes/no)	2.41	2.08–2.79	<0.0001
Endoscopic Barret’s mucosa ≥10 mm (yes/no)	2.62	1.71–4.04	<0.0001
Atrophic gastritis (yes/no)	0.40	0.34–0.47	<0.0001
Use of low-dose aspirin (yes/no)	0.38	0.17–0.76	0.0055

*OR*, odds ratio; *CI*, confidence interval; *BMI*, body mass index.

Since a gender difference was observed in the age distribution of the erosive esophagitis prevalence, we performed a multivariate analysis of factors associated with EE stratified by gender. As shown in S1 Table, the odds ratio for EE increased as the age group increased in the female subjects. Conversely, this relationship between age and EE was not observed in the male subjects, although the odds ratio was significantly higher for the 40- to 59- year-old group than for the ≤39-year-old group.

In addition, we performed a multivariate analysis of factors associated with EE in the subgroup subjects stratified by the presence of a hiatal hernia and/or endoscopic Barret’s mucosa. As shown in S2 Table, the factors associated with EE were similar regardless of the stratification of the study subjects.

Furthermore, we compared the clinical characteristics of the EE subjects and the control group, including subjects with missing STAI values, by bivariate analysis. As shown in S3 Table, the same factors that were significant in Table 2 differed significantly between the two groups, and the proportion of subjects with missing STAI values among the EE subjects was not significantly different from that among control individuals (4.4% (58/1320) vs. 5.5% (189/3443), *P* = 0.1270). Additionally, a multiple logistic regression analysis of the factors associated with EE compared to the control group was performed using three categories of STAI score (high STAI score, normal and low STAI score, and missing STAI score). As shown in S4 Table, the factors associated with EE were similar regardless of the inclusion of subjects with missing STAI values, and a missing STAI score was not a significant factor.

### Factors associated with symptomatic EE compared to mild symptomatic and asymptomatic EE

Among the 1,262 EE subjects, the proportions of subjects with sEE, msEE, and aEE were 15.0% (n = 189), 37.2% (n = 469) and 47.9% (n = 604), respectively. Table 4 shows comparisons of the clinical characteristics among the three EE groups. Alcohol consumption ≥20 g/day, sleep shortage, experiencing high levels of stress, feeling depressed, STAI score, EE ≥LA grade B, hiatal hernia of a severe grade, atrophic gastritis, and overlapping FD (PDS and EPS) differed significantly between the sEE group and the msEE plus aEE group (Tables 4 and S5).

**Table 4:** Comparison of the clinical characteristics of the three erosive esophagitis groups according to the frequency of reflux symptoms.

	sEE (n = 189)	msEE (n = 469)	aEE (n = 604)
Age, mean±SD (years)	52.2±9.3	52.3±9.3	52.9±9.6
Age group			
≤39 years (%)	7.4	8.7	7.3
40–59 years (%)	69.8	70.4	68.2
≥60 years (%)	22.8	20.9	24.5
Male (%)	86.8	84.7	82.3
BMI, mean±SD (kg/m^2^)	24.6±3.6	24.4±3.4	24.2±3.6
BMI ≥25 kg/m^2^ (%)	42.9	38.2	34.3
Current smoking (%)	26.5	24.7	24.5
Alcohol consumption ≥20 g /day (%)	45.5^a^	40.9	35.3
Sleep shortage (%)	37.6^a^	29.9	27.7
Exercise shortage (%)	68.3	70.8	59.6
Irregular meal time (%)	22.8	26.0	20.2
Experiencing high levels of stress (%)	45.0^b^	30.9	20.4
Feeling depressed (%)	14.3^b^	7.9	4.3
Kyphosis diagnosed by questionnaire (%)	1.1	0.64	0.66
STAI score, mean±SD	44.9±10.6^b^	41.5±10.1	39.7±9.1
High STAI score (%)	51.3^b^	37.7	32.8
Erosive esophagitis ≥LA grade B (%)	35.5^b^	22.0	14.2
Hiatal hernia severe grade (%)	18.0^b^	10.9	5.6
Endoscopic Barret’s mucosa ≥10 mm (%)	5.8	5.3	3.5
Atrophic gastritis (%)	31.8^a^	23.2	23.2
Use of NSAIDs (%)	1.6	1.7	1.7
Use of low-dose aspirin (%)	0.0	0.85	1.2
Use of Ca antagonists (%)	14.3	12.8	11.8
Use of ARB (%)	11.1	11.5	8.8
Use of statins (%)	10.1	11.3	10.4
Use of oral hypoglycemic agents (%)	4.8	4.3	5.8
Use of bisphosphonate (%)	0.0	0.21	0.17
Use of gastromucoprotective agents (%)	2.7	2.1	3.0
Overlapping with FD (%)	33.3^b^	1.5	1.2
PDS (%)	14.3^b^	0.64	0.83
EPS (%)	27.5^b^	0.85	0.33

*sEE*, symptomatic erosive esophagitis; *msEE*, mild symptomatic erosive esophagitis; *aEE*, asymptomatic erosive esophagitis; *SD*, standard deviation; *BMI*, body mass index; *STAI*, State-Trait Anxiety Inventory; *NSAIDs*, non-steroidal anti-inflammatory drugs; *Ca*, calcium; *ARB*, angiotensin II receptor blocker; *FD*, functional dyspepsia; *PDS*, postprandial distress syndrome; *EPS*, epigastric pain syndrome.

^a^ *P*<0.05 versus msEE plus aEE

^b^ *P*<0.0001 versus msEE plus aEE

In the multivariate analysis of factors associated with sEE compared to msEE plus aEE, overlapping EPS (OR 58.4, 95% CI 25.2–160.0), overlapping PDS (OR 9.96, 95% CI 3.91–26.8), hiatal hernia of a severe grade (OR 2.43, 95% CI 1.43–4.05), experiencing high levels of stress (OR 2.20, 95% CI 1.43–3.40), EE ≥LA grade B (OR 1.72, 95% CI 1.12–2.60), and atrophic gastritis (OR 1.57, 95% CI 1.03–2.36) were identified as significant factors (Table 5).

**Table 5:** Multivariate analysis of the factors associated with symptomatic erosive esophagitis compared to mild symptomatic plus asymptomatic erosive esophagitis.

	OR	95% CI	*P* value
Erosive esophagitis ≥LA grade B (yes/no)	1.72	1.12–2.60	0.0135
Hiatal hernia severe (yes/no)	2.43	1.43–4.05	0.0013
Atrophic gastritis (yes/no)	1.57	1.03–2.36	0.0353
Experiencing high levels of stress (yes/no)	2.20	1.43–3.40	0.0004
Overlapping with PDS (yes/no)	9.96	3.91–26.8	<0.0001
Overlapping with EPS (yes/no)	58.4	25.2–160.0	<0.0001

*OR*, odds ratio; *CI*, confidence interval; *BMI*, body mass index; *PDS*, postprandial distress syndrome; *EPS*, epigastric pain syndrome.

## Discussion

The results of the present study demonstrated that the prevalence of EE in subjects undergoing health checkups was 16.7%, and most EE cases (97.7%) were mild—grade A or B. In Japan, the prevalence of GERD has been increasing since the end of the 1990s [3,4]. One possible reason for this change is an increase in gastric acid secretion in the Japanese population, irrespective of *Helicobacter pylori* infection [18]. Since dietary fat intake has been reported to stimulate gastric acid secretion in mice [19], the chronological increase in gastric acid secretion in Japanese people may be related to an increase in fat intake [20]. In addition, because *H*. *pylori* infection is inversely related to the prevalence of GERD [3], a decrease in the *H*. *pylori* infection rate in the Japanese population is considered to be one explanation for this change.

Other than the absence of *H*. *pylori* infection, multiple risk factors for EE have been identified, including advanced age, male gender, BMI, smoking, alcohol consumption, the absence of gastric atrophy, endoscopic Barret’s mucosa, or hiatal hernia [6,21–26]. Consistent with previous studies, these factors were also significantly associated with EE compared to the control group in this study. In addition, in this study, experiencing high levels of stress was a significant risk factor associated with EE, which was consistent with the reported association between psychological factors and GERD [27]. Use of low-dose aspirin was a significant preventive factor associated with EE in the multivariate analysis, although it was not identified as a significant factor in the bivariate analysis. Because few low-dose aspirin users participated in this study, a coincidental statistical bias might exist in the analysis. An association between diabetes mellitus and GERD has been reported [28]. Unfortunately, because the fasting blood glucose and hemoglobin A1c values were not investigated in this study, we did not perform statistical analyses using diabetes mellitus as an explanatory variable. The use of oral hypoglycemic agents was a significant risk factor associated with EE in the bivariate analysis but was not a significant factor in the multivariate analysis.

Because it reduces patient QOL, the symptom presentation of EE is clinically important. Although the pathogenesis of reflux symptom manifestation in GERD is not fully understood, sensory nerve stimulation through direct contact with refluxed gastric acid is a primary factor. In addition, other factors, including esophageal visceral hypersensitivity, abnormal tissue resistance, or sustained esophageal contraction, might be involved in the presentation of reflux symptoms [29]. This study showed that the prevalence of sEE in EE subjects was 15.0%, and approximately half (47.9%) of the EE subjects were asymptomatic. In previous studies, the prevalence of sEE in EE subjects has ranged from 11.3% to 66.4% [9–11, 30], which is likely dependent upon the differences in definitions of sEE and study populations.

The significant independent predictors of sEE in this study included higher-grade EE (≥LA grade B), severe hiatal hernia, atrophic gastritis, experiencing high levels of stress, overlapping EPS, and overlapping PDS. The severities of esophagitis and hiatal hernia were associated with esophageal exposure to refluxed gastric acid, which suggests that the inhibition of acid is important for reflux symptom management.

Experiencing high levels of stress is considered a psychological factor. In the bivariate analysis in this study, other psychological stress-related factors, including sleep shortage, feeling depressed and STAI scores, were statistically significant risk factors associated with sEE. Previous studies have also shown that psychological factors, such as anxiety, depression, and somatization symptoms, were risk factors associated with sEE [9–11].

Dyspeptic symptoms are more common in patients with frequent GERD compared with intermittent GERD, and they impact patient QOL [31]. In agreement with previous studies [10,11], FD was a significant risk factor associated with sEE in this study. EPS showed a stronger association with sEE than PDS, which was also consistent with a previous study [10]. Patients with dyspepsia are known to be at risk to develop GERD [31]. In addition, specific dyspeptic symptoms are reportedly associated with poor responses to PPIs in GERD patients [32]. To better manage the reflux symptoms of EE, further pathophysiological studies of the overlap between GERD and FD are needed.

Atrophic gastritis, which is mainly induced by *H*. *pylori* infection, leads to hypoacidity of gastric juice and is negatively associated with EE. This negative association was also observed in this study. Interestingly, however, the presence of atrophic gastritis was also a risk factor associated with sEE in this study. A positive association between *H*. *pylori* infection and sEE has been reported in a previous study from China [30]. Chronic mucosal inflammation in atrophic gastritis might lead to abnormalities in gastroduodenal motility and sensitivity [33] as well as reflux symptom presentation.

One strength of this study was the use of a large catalog of data from a prospective multicenter cohort study. The size and comprehensiveness of this database enabled us to determine the predictors of EE and the factors associated with symptom presentation in EE while adjusting for potential confounders. Nevertheless, this study has several limitations. First, most of the EE subjects in this study were individuals who underwent health screenings, not patients. In addition, the severity of reflux symptoms was not measured, although symptom frequency was measured in detail. The questionnaire for self-assessment of daily life without an objective definition was simple, and therefore it might be insufficient for evaluating the data or for comparisons to other studies on the same topic. Subjects who were receiving antidepressants or tranquilizers were not excluded from this study, although their perceptions of their stress levels might be influenced by those drugs. Furthermore, the status of *H*. *pylori* infection was not evaluated biologically, although endoscopic atrophic gastritis associated with *H*. *pylori* infection was evaluated.

In conclusion, approximately one-sixth of the EE subjects in our population were symptomatic. The factors associated with the symptomatic presentation of EE included overlapping EPS, overlapping PDS, experiencing high levels of stress, severe hiatal hernia, atrophic gastritis, and LA grade B or worse. A multifactorial etiology, including factors unrelated to gastric acid secretion, was associated with the symptomatic presentation of EE. The natural history of EE symptom presentation will be investigated in our follow-up studies.

## Supporting information

S1 File Dataset of the subjects. (XLSX)

S1 Table Multivariate analysis of the factors associated with erosive esophagitis compared to the control group stratified by gender. (DOCX)

S2 Table Multivariate analysis of the factors associated with erosive esophagitis compared to the control group stratified by the presence of a hiatal hernia and endoscopic Barret’s mucosa. (DOCX)

S3 Table Comparison of clinical characteristics between the subjects with erosive esophagitis and control individuals (including the subjects with incomplete STAI values). (DOCX)

S4 Table Multivariate analysis of the factors associated with erosive esophagitis compared to the control group (including the subjects with incomplete STAI values). (DOCX)

S5 Table Comparison of clinical characteristics between the subjects with symptomatic erosive esophagitis and the subjects with mild symptomatic plus asymptomatic erosive esophagitis. (DOCX)
