# Supplementary material for: Factors associated with the presentation of erosive esophagitis symptoms in health checkup subjects: A prospective, multicenter cohort study
Source: PLoS One. 2018 May 3;13(5):e0196848. doi: 10.1371/journal.pone.0196848 (PMC5933688; doi:10.1371/journal.pone.0196848)
Supplement: S1 Table — (DOCX) [file pone.0196848.s002.docx]

**S1 Table. Multivariate analysis of the factors associated with erosive esophagitis compared to the control group stratified by gender.**

|  | OR | 95% CI | *P* value |
| --- | --- | --- | --- |
| Male |  |  |  |
| Age |  |  |  |
| 40-59 years (reference: ≤39 years) | 1.34 | 1.00-1.80 | 0.0493 |
| ≥60 years (reference: ≤39 years) | 1.17 | 0.84-1.64 | 0.3478 |
| BMI ≥25 kg/m^2^ (yes/no) | 1.72 | 1.45-2.05 | <0.0001 |
| Current smoking (yes/no) | 1.24 | 1.03-1.49 | 0.0261 |
| Alcohol consumption ≥20 g /day (yes/no) | 1.62 | 1.37-1.91 | <0.0001 |
| Experiencing high levels of stress (yes/no) | 1.33 | 1.07-1.64 | 0.0089 |
| Hiatal hernia (yes/no) | 2.43 | 2.07-2.86 | <0.0001 |
| Endoscopic Barret’s mucosa ≥10 mm (yes/no) | 2.59 | 1.62-4.16 | <0.0001 |
| Atrophic gastritis (yes/no) | 0.39 | 0.32-0.47 | <0.0001 |
| Use of low-dose aspirin (yes/no) | 0.49 | 0.22-0.99 | 0.0473 |
|  |  |  |  |
| Female |  |  |  |
| Age |  |  |  |
| 40-59 years (reference: ≤39 years) | 2.00 | 1.08-4.01 | 0.0258 |
| ≥60 years (reference: ≤39 years) | 3.70 | 1.81-8.08 | 0.0002 |
| BMI ≥25 kg/m^2^ (yes/no) | 2.70 | 1.84-3.95 | <0.0001 |
| Current smoking (yes/no) | 2.59 | 1.41-4.60 | 0.0026 |
| Alcohol consumption ≥20 g /day (yes/no) | 1.56 | 0.91-2.60 | 0.1034 |
| Experiencing high levels of stress (yes/no) | 1.61 | 1.10-2.36 | 0.0156 |
| Hiatal hernia (yes/no) | 2.33 | 1.65-3.28 | <0.0001 |
| Endoscopic Barret’s mucosa ≥10 mm (yes/no) | 2.67 | 0.83-7.91 | 0.0970 |
| Atrophic gastritis (yes/no) | 0.45 | 0.30-0.66 | <0.0001 |
| Use of gastromucoprotective agents (yes/no) | 3.56 | 1.37-9.09 | 0.0099 |

*OR,* odds ratio; *CI,* confidence interval; *BMI,* body mass index.
