# Supplementary material for: Factors associated with the presentation of erosive esophagitis symptoms in health checkup subjects: A prospective, multicenter cohort study
Source: PLoS One. 2018 May 3;13(5):e0196848. doi: 10.1371/journal.pone.0196848 (PMC5933688; doi:10.1371/journal.pone.0196848)
Supplement: S2 Table — (DOCX) [file pone.0196848.s003.docx]

**S2 Table. Multivariate analysis of the factors associated with erosive esophagitis compared to the control group stratified by the presence of a hiatal hernia and endoscopic Barret’s mucosa.**

|  | OR | 95% CI | *P* value |
| --- | --- | --- | --- |
| Hiatal hernia (+) and/or Barret’s mucosa (+) |  |  |  |
| Age |  |  |  |
| 40-59 years (reference: ≤39 years) | 1.67 | 1.12-2.50 | 0.0116 |
| ≥60 years (reference: ≤39 years) | 1.77 | 1.11-2.83 | 0.0153 |
| Gender (male/female) | 2.14 | 1.58-2.94 | <0.0001 |
| BMI ≥25 kg/m^2^ (yes/no) | 1.70 | 1.33-2.16 | <0.0001 |
| Current smoking (yes/no) | 1.31 | 1.00-1.71 | 0.0484 |
| Alcohol consumption ≥20 g /day (yes/no) | 1.86 | 1.46-2.38 | <0.0001 |
| Experiencing high levels of stress (yes/no) | 1.34 | 1.01-1.80 | 0.0446 |
| Hiatal hernia severe (yes/no) | 2.09 | 1.51-2.91 | <0.0001 |
| Endoscopic Barret’s mucosa ≥10 mm (yes/no) | 1.68 | 1.09-2.60 | 0.0190 |
| Atrophic gastritis (yes/no) | 0.38 | 0.29-0.49 | <0.0001 |
| Use of low-dose aspirin (yes/no) | 0.29 | 0.10-0.73 | 0.0080 |
|  |  |  |  |
| Hiatal hernia (-) and Barret’s mucosa (-) |  |  |  |
| Age |  |  |  |
| 40-59 years (reference: ≤39 years) | 1.33 | 0.95-1.89 | 0.0954 |
| ≥60 years (reference: ≤39 years) | 1.41 | 0.95-2.10 | 0.0873 |
| Gender (male/female) | 2.43 | 1.93-3.07 | <0.0001 |
| BMI ≥25 kg/m^2^ (yes/no) | 2.02 | 1.64-2.49 | <0.0001 |
| Current smoking (yes/no) | 1.37 | 1.08-1.74 | 0.0103 |
| Alcohol consumption ≥20 g /day (yes/no) | 1.40 | 1.14-1.73 | 0.0015 |
| Experiencing high levels of stress (yes/no) | 1.38 | 1.09-1.75 | 0.0084 |
| Atrophic gastritis (yes/no) | 0.40 | 0.32-0.50 | <0.0001 |
| Use of low-dose aspirin (yes/no) | 0.48 | 0.13-1.35 | 0.1770 |

*OR,* odds ratio; *CI,* confidence interval; *BMI,* body mass index.
