# Supplementary material for: Factors associated with the presentation of erosive esophagitis symptoms in health checkup subjects: A prospective, multicenter cohort study
Source: PLoS One. 2018 May 3;13(5):e0196848. doi: 10.1371/journal.pone.0196848 (PMC5933688; doi:10.1371/journal.pone.0196848)
Supplement: S3 Table — (DOCX) [file pone.0196848.s004.docx]

S3 Table. Comparison of clinical characteristics between the subjects with erosive esophagitis and control individuals (including the subjects with incomplete STAI values).

|  | EE  (n=1,320) | Control (n=3,443) | *P* value |
| --- | --- | --- | --- |
| Age, mean±SD (years) | 52.7±9.5 | 53.0±10.4 | 0.4496 |
| Age group |  |  | 0.0076 |
| ≤39 years (%) | 7.7 | 9.2 |  |
| 40-59 years (%) | 68.3 | 63.5 |  |
| ≥60 years (%) | 24.0 | 27.3 |  |
| Male (%) | 83.7 | 61.4 | <0.0001 |
| BMI, mean±SD | 24.3±3.6 | 22.7±3.1 | <0.0001 |
| BMI ≥25 kg/m^2^ (%) | 36.8 | 20.4 | <0.0001 |
| Current smoking (%) | 24.8 | 15.1 | <0.0001 |
| Alcohol consumption ≥20 g /day (%) | 38.9 | 23.6 | <0.0001 |
| Sleep shortage (%) | 29.9 | 28.5 | 0.3194 |
| Exercise shortage (%) | 64.7 | 60.7 | 0.0111 |
| Irregular meal time (%) | 22.4 | 19.1 | 0.0113 |
| Experiencing high levels of stress (%) | 27.8 | 20.2 | <0.0001 |
| Feeling depressed (%) | 7.1 | 5.5 | 0.0460 |
| Kyphosis diagnosed by questionnaire (%) | 0.68 | 0.35 | 0.1202 |
| STAI score, mean±SD | 41.1±9.9 | 40.0±9.5 | 0.0004 |
| High STAI score (%) | 35.8 | 29.6 | <0.0001 |
| Missing STAI score (%) | 4.4 | 5.5 | 0.1270 |
| Hiatal hernia (%) | 48.2 | 24.1 | <0.0001 |
| Endoscopic Barret’s mucosa ≥10 mm (%) | 4.5 | 1.5 | <0.0001 |
| Atrophic gastritis (%) | 24.6 | 41.1 | <0.0001 |
| Use of NSAIDs (%) | 1.7 | 1.3 | 0.2231 |
| Use of low-dose aspirin (%) | 1.1 | 1.4 | 0.4029 |
| Use of Ca antagonists (%) | 12.5 | 9.1 | 0.0004 |
| Use of ARB (%) | 10.1 | 7.4 | 0.0026 |
| Use of statins (%) | 10.5 | 9.5 | 0.2971 |
| Use of oral hypoglycemic agents (%) | 5.1 | 3.3 | 0.0031 |
| Use of bisphosphonate (%) | 0.15 | 0.38 | 0.2619 |
| Use of gastromucoprotective agents (%) | 2.7 | 2.0 | 0.1281 |

*EE,* erosive esophagitis; *SD,* standard deviation; *BMI,* body mass index; *STAI,* State-Trait Anxiety Inventory; *NSAIDs,* non-steroidal anti-inflammatory drugs; *Ca,* calcium; *ARB,* angiotensin II receptor blocker.
