# Supplementary material for: Factors associated with the presentation of erosive esophagitis symptoms in health checkup subjects: A prospective, multicenter cohort study
Source: PLoS One. 2018 May 3;13(5):e0196848. doi: 10.1371/journal.pone.0196848 (PMC5933688; doi:10.1371/journal.pone.0196848)
Supplement: S4 Table — (DOCX) [file pone.0196848.s005.docx]

**S4 Table. Multivariate analysis of the factors associated with erosive esophagitis compared to the control group (including the subjects with incomplete STAI values).**

|  | OR | 95% CI | *P* value |
| --- | --- | --- | --- |
| Age |  |  |  |
| 40-59 years (reference: ≤39 years) | 1.44 | 1.12-1.87 | 0.0044 |
| ≥60 years (reference: ≤39 years) | 1.55 | 1.15-2.09 | 0.0035 |
| Gender (male/female) | 2.34 | 1.95-2.80 | <0.0001 |
| BMI ≥25 kg/m^2^ (yes/no) | 1.84 | 1.58-2.15 | <0.0001 |
| Current smoking (yes/no) | 1.31 | 1.10-1.56 | 0.0021 |
| Alcohol consumption ≥20 g /day (yes/no) | 1.58 | 1.35-1.84 | <0.0001 |
| Experiencing high levels of stress (yes/no) | 1.44 | 1.20-1.72 | <0.0001 |
| Hiatal hernia (yes/no) | 2.44 | 2.12-2.82 | <0.0001 |
| Endoscopic Barret’s mucosa ≥10 mm (yes/no) | 2.51 | 1.66-3.83 | <0.0001 |
| Atrophic gastritis (yes/no) | 0.39 | 0.33-0.46 | <0.0001 |
| Use of low-dose aspirin (yes/no) | 0.47 | 0.24-0.90 | 0.0215 |
| High STAI score (reference: normal and low STAI score) | 1.08 | 0.92-1.27 | 0.3620 |
| Missing STAI score (reference: normal and low STAI score) | 0.91 | 0.65-1.26 | 0.5751 |

*OR,* odds ratio; *CI,* confidence interval; *BMI,* body mass index; *STAI,* State-Trait Anxiety Inventory.
