# Supplementary material for: Factors associated with the presentation of erosive esophagitis symptoms in health checkup subjects: A prospective, multicenter cohort study
Source: PLoS One. 2018 May 3;13(5):e0196848. doi: 10.1371/journal.pone.0196848 (PMC5933688; doi:10.1371/journal.pone.0196848)
Supplement: S5 Table — (DOCX) [file pone.0196848.s006.docx]

**S5 Table. Comparison of clinical characteristics between the subjects with symptomatic erosive esophagitis and the subjects with mild symptomatic plus asymptomatic erosive esophagitis.**

|  | sEE (n=189) | msEE +aEE (n=1073) | *P* value |
| --- | --- | --- | --- |
| Age, mean±SD (years) | 52.2±9.3 | 52.6±9.4 | 0.5866 |
| Age group |  |  | 0.9669 |
| ≤39 years (%) | 7.4 | 7.9 |  |
| 40-59 years (%) | 69.8 | 69.2 |  |
| ≥60 years (%) | 22.8 | 22.9 |  |
| Male (%) | 86.8 | 83.3 | 0.2342 |
| BMI (mean±SD) | 24.6±3.6 | 24.3±3.6 | 0.3359 |
| BMI ≥25 kg/m^2^ (%) | 42.9 | 36.0 | 0.0707 |
| Current smoking (%) | 26.5 | 24.6 | 0.5873 |
| Alcohol consumption ≥20 g /day (%) | 45.5 | 37.7 | 0.0437 |
| Sleep shortage (%) | 37.6 | 28.6 | 0.0132 |
| Exercise shortage (%) | 68.3 | 64.5 | 0.3172 |
| Irregular meal time (%) | 22.8 | 22.7 | 0.9973 |
| Experiencing high levels of stress (%) | 45.0 | 25.0 | <0.0001 |
| Feeling depressed (%) | 14.3 | 5.9 | <0.0001 |
| Kyphosis diagnosed by questionnaire (%) | 1.1 | 0.65 | 0.6310 |
| STAI score, mean±SD | 44.9±10.6 | 40.4±9.6 | <0.0001 |
| High STAI score (%) | 51.3 | 35.0 | <0.0001 |
| Erosive esophagitis ≥LA grade B (%) | 35.5 | 17.6 | <0.0001 |
| Hiatal hernia severe grade (%) | 18.0 | 7.9 | <0.0001 |
| Endoscopic Barret’s mucosa ≥10 mm (%) | 5.8 | 4.3 | 0.3494 |
| Atrophic gastritis (%) | 31.8 | 23.2 | 0.0118 |
| Use of NSAIDs (%) | 1.6 | 1.7 | 1.000 |
| Use of low-dose aspirin (%) | 0.0 | 1.0 | 0.3864 |
| Use of Ca antagonists (%) | 14.3 | 12.2 | 0.4263 |
| Use of ARB (%) | 11.1 | 10.0 | 0.6324 |
| Use of statins (%) | 10.1 | 10.8 | 0.7559 |
| Use of oral hypoglycemic agents (%) | 4.8 | 5.1 | 0.8335 |
| Use of bisphosphonate (%) | 0.0 | 0.19 | 1.000 |
| Use of gastromucoprotective agents (%) | 2.7 | 2.6 | 1.000 |
| Overlapping with FD (%) | 33.3 | 1.3 | <0.0001 |
| PDS (%) | 14.3 | 0.75 | <0.0001 |
| EPS (%) | 27.5 | 0.56 | <0.0001 |

*EE,* erosive esophagitis; *SD,* standard deviation; *BMI,* body mass index; *STAI,* State-Trait Anxiety Inventory; *NSAIDs,* non-steroidal anti-inflammatory drugs; *Ca,* calcium; *ARB,* angiotensin II receptor blocker.
